# Immunotherapy in gestational trophoblastic neoplasia: advances and future directions

**DOI:** 10.3389/fimmu.2025.1544585

**Published:** 2025-04-11

**Authors:** Jing Zeng, Jing Zhang, Jianzhang Wang, Lian Xu, Cheng Wang, Rutie Yin

**Affiliations:** ^1^ Department of Gynecology and Obstetrics, West China Second University Hospital, Sichuan University, Chengdu, Sichuan, China; ^2^ Key Laboratory of Birth Defects and Related Diseases of Women and Children, Ministry of Education, Chengdu, Sichuan, China; ^3^ Joint Laboratory of Reproductive Medicine (SCU-CUHK), West China Second University Hospital, Sichuan University, Chengdu, Sichuan, China; ^4^ West China School of Medicine, Sichuan University, Chengdu, Sichuan, China; ^5^ Department of Pathology, West China Second University Hospital, Sichuan University, Chengdu, Sichuan, China

**Keywords:** gestational trophoblastic neoplasia, immune checkpoint inhibitors, chemoresistance, clinical trials, fertility preservation, immune-related adverse events

## Abstract

Gestational trophoblastic neoplasia (GTN) is a rare but aggressive malignancy that follows normal or aberrant pregnancies. Until the advent of immunotherapy in 2017, surgery and chemotherapy were the standard treatment modalities, with chemotherapy remaining the cornerstone. However, chemoresistance and high-risk disease present significant challenges in managing GTN. Recent advancements in immunotherapy, particularly immune checkpoint inhibitors (ICIs), have offered new hope for managing these difficult cases. This review provides the comprehensive overview of the mechanisms underlying ICIs in GTN, and explores the potential synergy of combining ICIs with targeted therapies, such as vascular endothelial growth factor and epidermal growth factor receptor inhibitors. We also provide an overview of the latest evidence on the use of ICIs in treating GTN, focusing on their effectiveness in both low- and high-risk cases, as well as in chemorefractory settings. In addition, we discuss ongoing clinical trials, immune-related adverse events associated with ICIs, biomarker-driven approaches, immunosuppressive tumor microenvironments, and the challenges posed with ICIs resistance. The review also explores future directions, including the integration of ICIs into standard regimens, the potential for personalized treatment based on tumor biology, and the importance of fertility preservation in young patients with GTN. In conclusion, while challenges remain, immunotherapy represents a promising frontier in GTN treatment, with the potential to improve outcomes and provide a more personalized approach to care

## 1 Introduction

**Figure 1:**
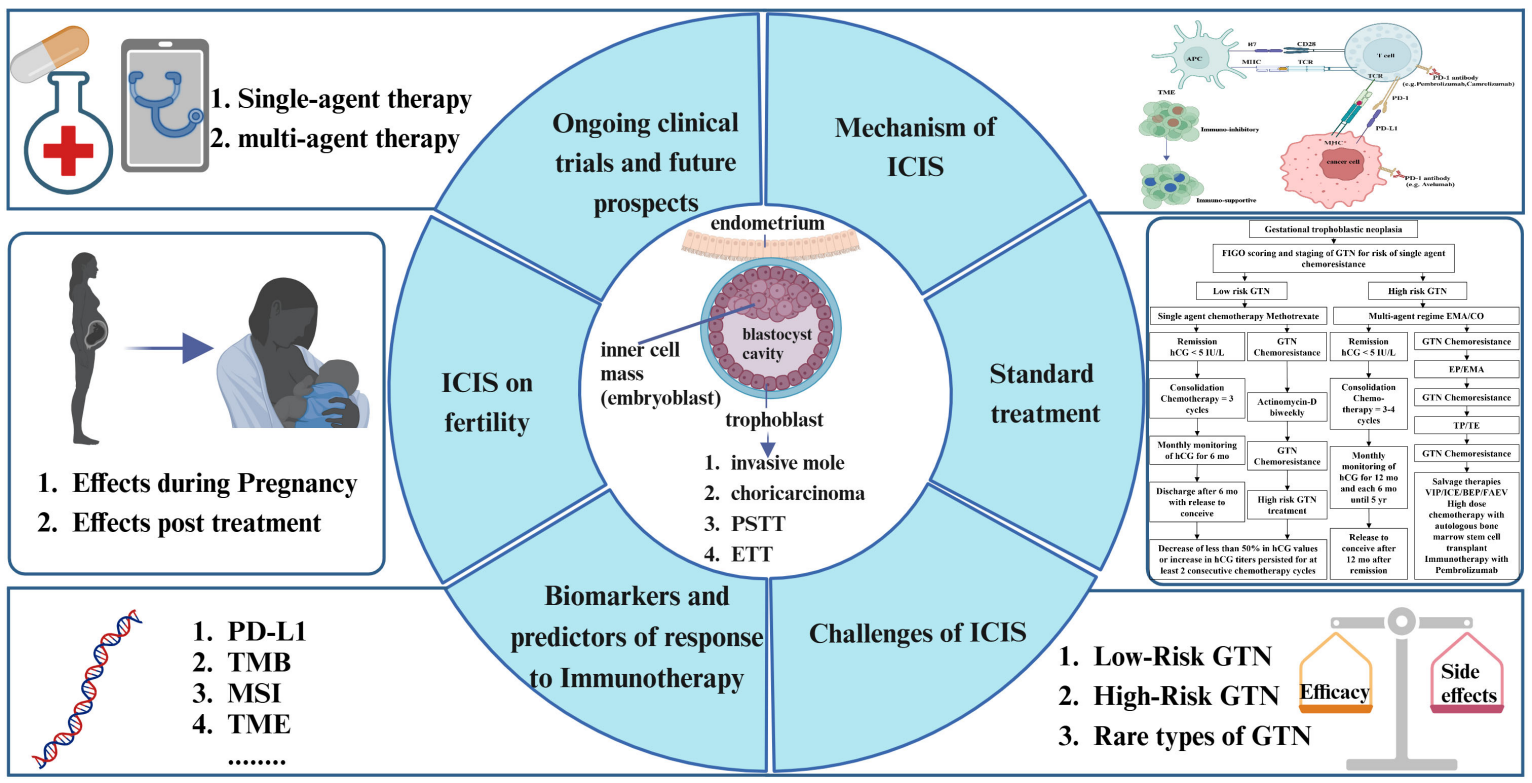
Conceptual framework of our study. This figure outlines the comprehensive approach to investigating immune checkpoint inhibitors (ICIs) in gestational trophoblastic neoplasia (GTN). It encompasses the mechanisms of ICIs in immune regulation, therapeutic efficacy, integration with standard treatments, exploration of predictive biomarkers, fertility-related considerations, and forward-looking perspectives on the future of GTN immunotherapy.

Gestational trophoblastic neoplasia (GTN) represents a rare yet distinct category of malignancies originating from the abnormal transformation of placental trophoblastic tissues following normal or aberrant fertilization. It encompasses various tumor types, including invasive mole, choriocarcinoma, placental site trophoblastic tumor (PSTT), and epithelioid trophoblastic tumor (ETT) (1). According to the International Federation of Gynecology and Obstetrics (FIGO) 2000 risk scoring system, GTN is stratified into low-risk (FIGO score <7), high-risk (FIGO score ≥7), and ultra-high-risk (FIGO score ≥13) groups. However, PSTT and ETT are not managed based on the FIGO score (2). While low-risk GTN typically responds well to single-agent chemotherapy, high-risk GTN requires multi-agent chemotherapy (1). However, approximately 5% of patients experience chemotherapy resistance or relapse, highlighting an unmet need for novel therapeutic strategies (3–5). Immunotherapy has emerged as a promising option, especially for chemoresistant GTN. In particular, immune checkpoint inhibitors (ICIs) targeting the programmed cell death protein 1/programmed cell death ligand 1(PD-1/PD-L1) axis have demonstrated notable efficacy in cases where conventional chemotherapy fails. This review examines advances in immunotherapy for GTN, focusing on its mechanisms, clinical efficacy, Immune-related adverse events (irAEs) with ICIs, impact on fertility, and future research directions (**Figure 1**).

## 2 Standard treatment in GTN

### 2.1 Treatment in low-risk GTN

**Figure 2:**
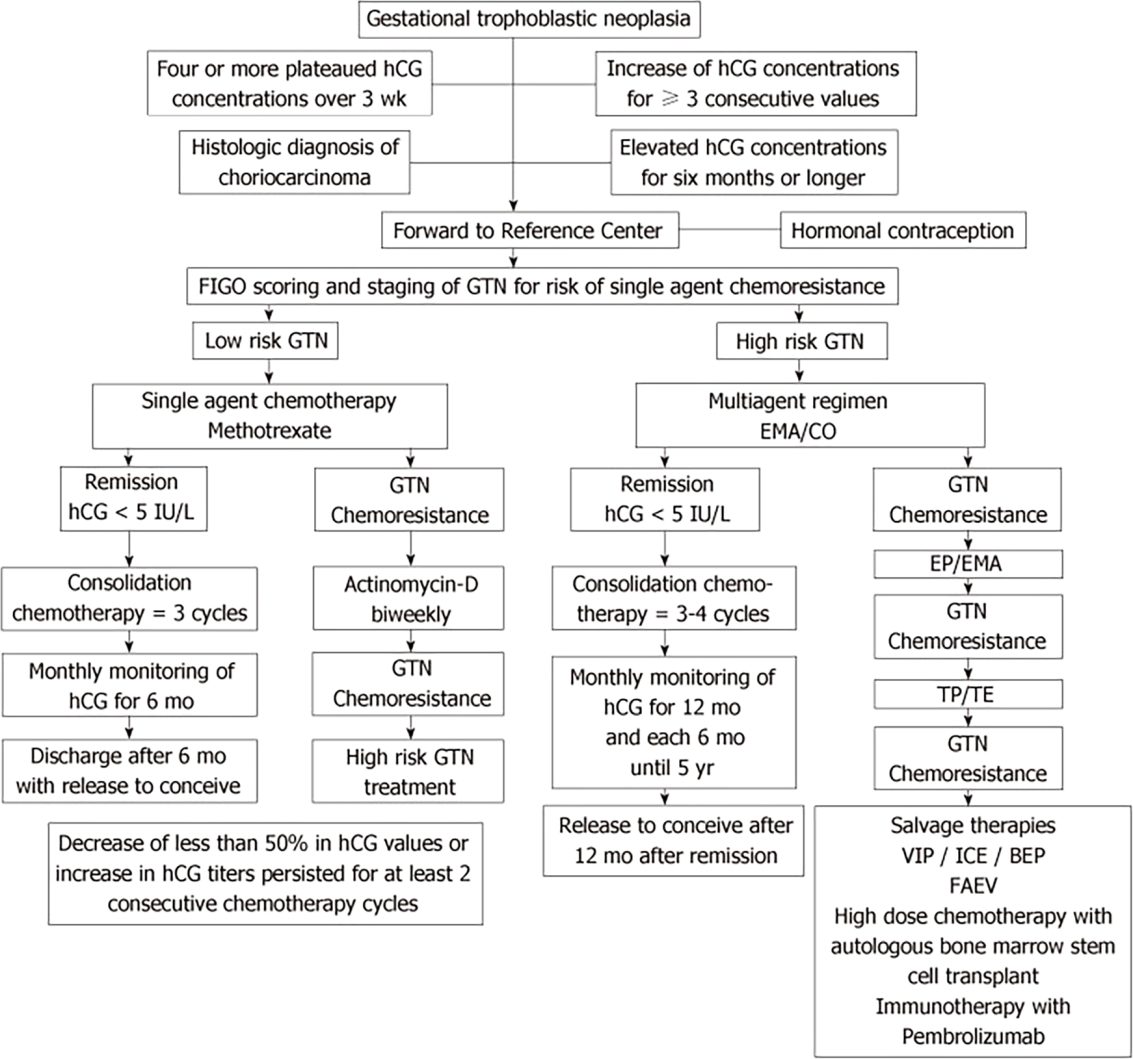
Algorithm for standard treatment of gestational trophoblastic neoplasia [adapted from Braga et al., (6)].

The standard treatment of GTN is stratified into low-risk and high-risk categories based on the FIGO scoring system, which informs the choice of therapeutic strategy (6). (**Figure 2**) As above, ultra-high risk and PSTT/ETT are treated separately. Low-risk GTN (FIGO score <7) typically responds well to single-agent chemotherapy. For women who have completed childbearing and do not have metastatic disease, then a hysterectomy could be considered (7). Commonly used agents include methotrexate and folinic acid (MTX/FA) or actinomycin-D (ActD), both showing high remission rates with minimal toxicity. MTX, which can be administered as a weekly intramuscular injection or in multi-day protocols, is often chosen for its high efficacy and manageable toxicity profile. ActD serves as an alternative, especially for patients who do not respond well to MTX or experience unacceptable side effects (8). Patients scoring 0–1 on the FIGO scale have a high cure rate (90%) with single-agent therapy, but resistance risk rises in patients with scores of 5–6, with only one-third achieving a cure with initial single-agent treatment (9). Resistance to MTX/FA can often be managed successfully by switching to ActD. Multi-agent regimens like EMA/CO (etoposide, methotrexate, and actinomycin-D/cyclophosphamide and vincristine) are reserved for patients with higher hCG levels or when initial single-agent therapies fail. Recent practices have adjusted the hCG threshold for transitioning to multi-agent treatments, with centers like Charing Cross hospital using a threshold up to 3000 IU/l to reduce the need for EMA/CO, minimizing toxicity (10).

### 2.2 Treatment in high-risk GTN

Women with a FIGO score of 7 or higher receive multi-agent chemotherapy, typically using the EMA/CO regimen. For patients with a score of 13 or higher, considered “ultra-high risk,” there is a significant risk of early and late mortality due to complications like hemorrhage or metabolic issues from a heavy tumor burden. Low-dose etoposide and cisplatin induction therapy can mitigate early death (11, 12). For those with liver metastases, EP/EMA regimen (etoposide, cisplatin/etoposide, methotrexate, actinomycin-D) may reduce the risk of late deaths from drug resistance. Treatment continues with EMA/CO until hCG normalization, followed by consolidation therapy for 6–8 weeks, particularly if high-risk features (e.g., brain or liver metastases) are present. Studies evaluating alternative regimens like 4-day MEA (etoposide, methotrexate, actinomycin-D) or FAEV(5-fluorouracil, actinomycin D, etoposide, vincristine) have shown activity but without clear superiority over EMA/CO (13, 14).

### 2.3 Treatment in chemoresistant or relapsed GTN

For patients with chemoresistant or relapsed GTN, there are no randomized trials specifically evaluating treatment options for GTN relapse, and existing studies often do not differentiate between resistance and relapse, indicating that the same chemotherapy regimens used for chemoresistant cases may also apply to relapsed disease. Prior to treatment, all patients should be re-staged to identify new metastatic sites. Surgical resection of isolated metastatic lesions is advised, and Positron Emission Tomography/Computed Tomography (PET/CT) can help differentiate viable tumor from necrotic tissue. For high-risk relapsed cases, sequential multi-agent regimens such as EMA-EP or TP-TE (paclitaxel, cisplatin/paclitaxel, etoposide) have shown better outcomes, but if resistance occurs, options like BEP (bleomycin, etoposide, cisplatin) or ICE (ifosfamide, carboplatin, cisplatin) regimens may be used (6, 15, 16). Immunotherapy, particularly ICIs, offers a promising alternative for chemoresistant GTN, with emerging evidence supporting its efficacy.

### 2.4 Treatment in rare type GTN

PSTT and ETT present unique challenges as they typically show less responsiveness to conventional chemotherapy. These subtypes often require surgical intervention, such as hysterectomy, especially for localized stage I disease, which can achieve high long-term survival rates (17). In cases of advanced or metastatic disease, platinum-based multi-agent regimens, such as EP/EMA and TP/TE are commonly used, although the prognosis remains challenging with overall survival rates between 50% and 60% (17, 18). It is important to note that an interval ≥48 months from their last known pregnancy and stage IV disease are important predictors of chemoresistance (17, 19). For patients with chemotherapy-resistant disease, newer treatments like ICIs have shown promise in achieving complete remission (20). Careful monitoring of prognostic factors, including FIGO stage, hCG levels, and time from the antecedent pregnancy, is crucial in guiding treatment decisions. The use of adjuvant chemotherapy is also an important consideration in these cases. Referral to specialized centers is recommended for high-risk cases to ensure optimal management.

In summary, the standard treatment for GTN involves a tailored approach based on the patient’s risk classification, ranging from single-agent chemotherapy for low-risk cases to multi-agent regimens and surgical interventions for high-risk and chemoresistant forms. Emerging targeted therapies and immunotherapies hold promise for improving outcomes, especially in cases where traditional treatments fail.

## 3 Mechanisms related to GTN treatment

### 3.1 Different subtypes of GTN

GTN encompasses four tumor types: invasive mole, choriocarcinoma, PSTT, and ETT, all of which originate from abnormal trophoblastic proliferation. These subtypes exhibit distinct genetic, epigenetic, and clinical characteristics that influence their behavior and treatment responses. Invasive moles, characterized by the penetration of molar villi into the myometrium, originate from complete or partial hydatidiform moles. Partial hydatidiform moles are typically triploid, containing both maternal and paternal genetic material, while complete hydatidiform moles are usually diploid, consisting only of paternal genes due to the absence of maternal DNA (16). Choriocarcinoma is a highly malignant tumor arising from any gestational event, characterized by abnormal trophoblastic hyperplasia and anaplasia, the absence of chorionic villi and vascular invasion. Its aggressiveness is often linked to epigenetic modifications, such as hypermethylation of tumor suppressor genes like DNA methyltransferase 3 beta (DNMT3B), which is the enzyme involved in *de novo* methylation of DNA during development (21). The process of hypermethylation significantly influences key molecular pathways, including phosphatidylinositol 3-hydroxy kinase-Akt (PI3K-Akt), human epidermal growth factor receptor-2 (ERBB2)/ERBB2 receptor tyrosine kinase 3 (ERBB3), and Janus kinase- signal transducer and activator of transcription (JAK-STAT), by promoting cellular proliferation, enhancing migration, and enabling the evasion of apoptotic signals (21). However, the pathways altered in choriocarcinoma remain poorly understood, and available data are limited.

Based on limited data, PSTT and ETT are thought to originate from different subtyped of trophoblast (22). The precise cell of origin remains speculative and poorly understood. While different trophoblast subtypes, including villous cytotrophoblasts, syncytiotrophoblasts, and intermediate trophoblasts, play distinct roles during normal placental development (23), the relationship between these cell types and the malignancy of GTN remains unclear. Disease biology may be related to the cell of origin, but this requires further investigation and clarification. Compared to choriocarcinoma, PSTT and ETT exhibit slower growth and lower hCG levels, along with chemotherapy resistance. These tumors are generally diploid with biparental contributions. The presence of both maternal and paternal genetic material, or in the case of a complete hydatidiform mole, exclusively paternal DNA, renders GTN highly immunogenic and particularly susceptible to immunotherapeutic interventions.

### 3.2 Mechanism of ICIs in GTN

The immune microenvironment in GTN shares similarities with the placental environment, characterized by the presence of regulatory T cells (Tregs) and immunosuppressive cytokines like Interleukin-10 (IL-10) and transforming growth factor-β (TGF-β), which create an anti-inflammatory state that supports tumor survival (24). These factors inhibit cytotoxic immune responses, aiding in tumor persistence. The immunosuppressive environment in GTN is further reinforced by the expression of indoleamine 2,3-dioxygenase (IDO), which modulates tryptophan metabolism, leading to T cell suppression and heightened immune tolerance (24).

**Figure 3:**
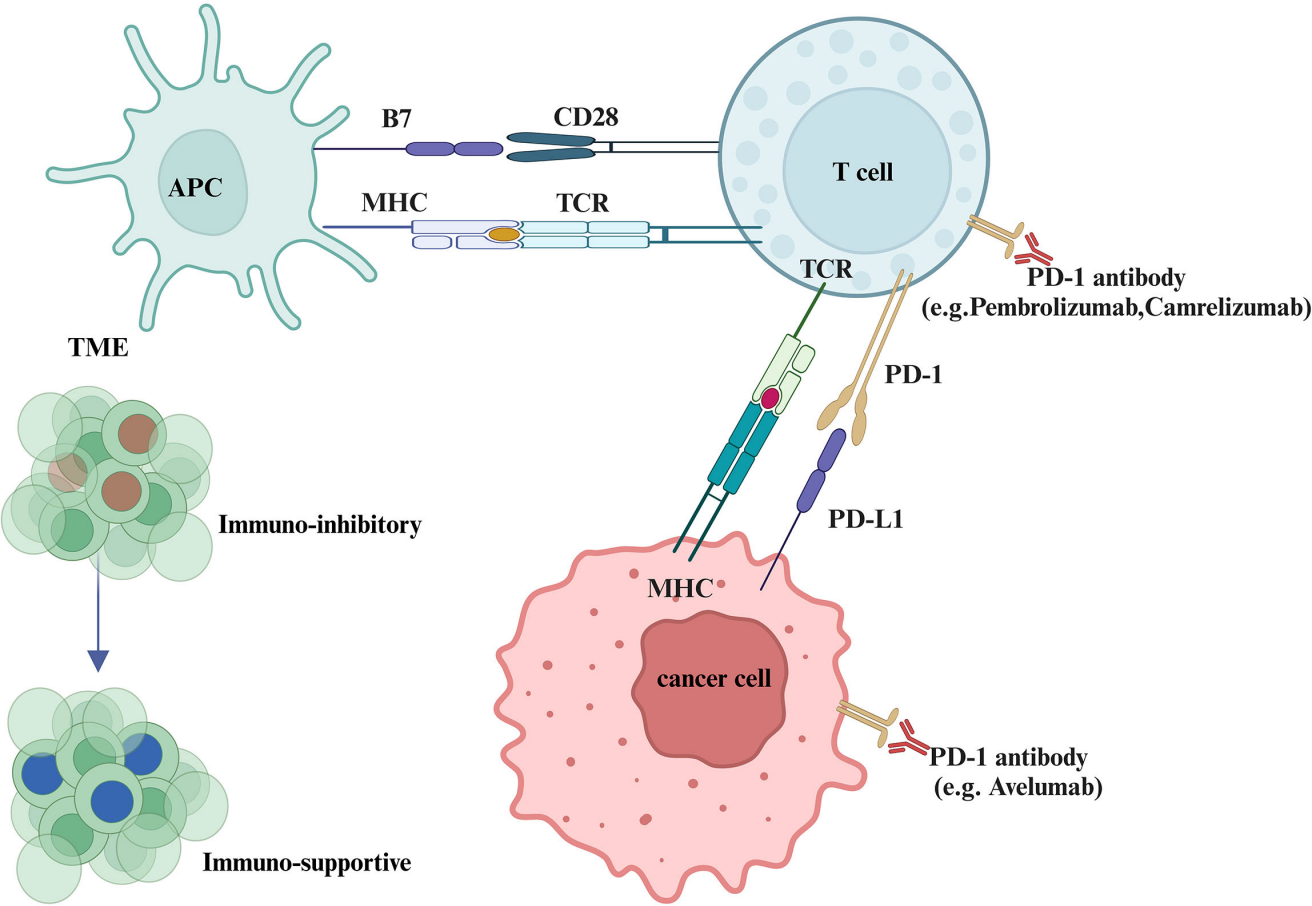
Pathogenesis of PD-1/PD-L1 blockade in malignant tumors. This figure illustrates how PD-1/PD-L1 interactions facilitate tumor immune evasion. Tumor cells suppress cytotoxic T lymphocytes (CTLs) by expressing co-inhibitory molecules such as PD-L1, leading to immune escape. When PD-1 antibodies (e.g., pembrolizumab, camrelizumab) or PD-L1 inhibitors (e.g., avelumab) are administered, CTLs regain their tumor-killing capabilities.The figure also highlights how the tumor immune microenvironment (TME) from an immunosuppressive to an activated state, which is enriched with immunosuppressive factors like regulatory T cells (Tregs) and cytokines such as TGF-β and IL-10, can inhibit immune responses and support tumor survival. ICIs can shift the TME toward activation by promoting the activity of tumor-infiltrating lymphocytes (TILs), especially CD8+ cytotoxic T cells.

Tumor immune escape plays a critical role in the malignancy of GTN, with immune checkpoints serving as key mechanisms that prevent apoptosis of cancer cells. PD-1, expressed predominantly on activated cytotoxic T lymphocytes (CTLs), is upregulated during T cell activation, not just under chronic antigen stimulation, such as cancer. The upregulation is asscociated with reduced T cell function, which impairs the immune system’s ability to effectively target and eliminate tumor cells (25–27). Tumor cells exploit this by expressing co-inhibitory molecules like members of the B7 family, suppressing CTLs and evading immune detection (26, 28). Immunohistochemistry studies show that PD-L1 is strongly expressed in various GTN cell types, including syncytiotrophoblasts and cytotrophoblasts, indicating that GTN employs immune evasion strategies similar to the placenta (29). The high levels of PD-L1 expression in GTN make PD-1/PD-L1 inhibitors a logical therapeutic approach. Studies have shown that blocking PD-1/PD-L1 signaling with drugs like Pembrolizumab can restore immune surveillance, leading to tumor regression in patients with chemoresistant GTN (30, 31). Additionally, other B7 family members, such as B7-H3 (CD276) and VISTA (PD-H1), are also highly expressed in both normal placental tissue and GTN. While VISTA’s exact receptor remains unknown, other B7 family members expression, particularly when occurring alongside PD-L1, further supports the rationale for targeting the PD-1/PD-L1 pathway with ICIs as a potential treatment not only for chemo-resistant GTN but also for patients with newly diagnosed disease who are at high risk of developing resistance (29, 32). **Figure 3** shows the pathogenesis of PD-1/PD-L1 blockade in GTN.

### 3.3 Mechanisms of combined therapy in GTN

Combining ICIs with targeted therapies that inhibit pathways like vascular endothelial growth factor (VEGF) and epidermal growth factor receptor (EGFR) may further enhance efficacy by addressing tumor angiogenesis and proliferation. VEGF promotes tumor angiogenesis, supporting tumor growth and metastasis, and contributes to an immunosuppressive tumor microenvironment by recruiting regulatory Tregs and inhibiting dendritic cell maturation, which impairs antigen presentation and reduces T cell activation (33). Combining VEGF-targeted therapies with immune ICIs, such as those blocking the PD-1/PD-L1 pathway, has shown potential to enhance anti-tumor responses (34). VEGF inhibition can decrease the immunosuppressive properties of the tumor microenvironment, thereby supporting T cell activity and improving the efficacy of ICIs (33). This combined approach reduces tumor vascular support while boosting immune system engagement, providing a potential strategy for treating aggressive and chemoresistant GTN. EGFR, part of the ERBB receptor family, is also overexpressed in GTN and is associated with increased cell proliferation and survival (35). Research indicates that drugs such as lapatinib can inhibit GTN cell growth by modulating EGFR and ERBB2 activity (36). These pathways underscore the potential of integrating targeted therapies with immunotherapy to improve GTN treatment outcomes.

## 4 Clinical efficacy of ICIs

### 4.1 Application of ICIs in low-risk GTN

Low-risk GTN generally responds well to single-agent chemotherapy, with cure rates exceeding 90%. For this reason, ICIs have not been widely utilized in low-risk GTN, as standard treatments are highly effective. However, in cases where patients are intolerant to chemotherapy, or when chemotherapy poses significant risk due to toxicity, the exploration of alternative therapies like ICIs has emerged as a valuable avenue of research. One significant investigation into the role of ICIs in GTN, particularly low-risk cases, is cohort A of the TROPHIMMUN Phase II Trial (37). **Table 1** shows the key phase II clinical trials of immunotherapy for GTN.

**Table 1:** Key phase II clinical trials of immunotherapy for gestational trophoblastic neoplasia.

Study Name	Agents	Participants	Complete response (N, %)	Duration of follow-up, months, median (IQR)	Grade 3* TRAEs (N, %)
TROPHIMMUN arm A (N=15)	Avelumab	1. GTN resistant to single agent chemotherapy 2. Any number of previous lines of chemotherapy	8 (53.3)	25 (NA)	0
TROPHIMMUN arm B (N =7)	Avelumab	1. GTN resistant to combination chemotherapy 2. Any number of previous lines of chemotherapy	1 (14.3)	18.2 (NA)	0
CAP-01 (N =20)	Camrelizumab and apatinib	1. Resistant/relapsed GTN following multidrug chemotherapy 2.Received at least 2 lines of multidrug chemotherapy	10 (50)	18.5 (14.6–20.9)	16 (80%)

N, number; IQR, interquartile range; TRAEs, treatment related adverse events; GTN, gestational trophoblastic neoplasia; NA, not available; *Grade 3 adverse events defined according to the National Cancer Institute Common Terminology Criteria for Adverse Events version 4.0 and 5.0.

The TROPHIMMUN Phase II Trial is a pivotal study evaluating the efficacy and safety of Avelumab, an anti-PD-L1 immune checkpoint inhibitor, in GTN patients. The trial includes two cohorts: Cohort A, focused on low-risk GTN patients first-line treatment resistant to MTX, and Cohort B, targeting high-risk or chemoresistant GTN (37, 38). In Cohort A, 15 patients were treated with avelumab at a dose of 10 mg/kg bi-weekly until normalization of serum hCG levels, followed by three consolidation cycles. Results showed that 8 patients (53.3%) achieved complete remission with normalized hCG levels after a median of 9 treatment cycles, and none experienced relapse after a median follow-up of 29 months. The remaining 7 patients required further chemotherapy or surgery to achieve remission. Avelumab demonstrated a favorable safety profile, with most treatment-related adverse events (TRAEs) being mild (grade 1-2), including fatigue and infusion-related reactions (37). The findings suggest that avelumab could be an alternative for chemotherapy-resistant GTN, especially for patients preferring to avoid traditional side effects or those intolerant to MTX or ActD.

However, other scholars have pointed out Avelumab achieved a 53% remission rate, which falls short compared to the 75% remission rate observed with second-line ActD (39, 40). ActD remains the preferred option due to its proven efficacy. Additionally, while ICIs are more costly and long-term safety data are lacking, chemotherapy continues to demonstrate high success rates with manageable adverse effects. This underscores that, although ICIs provide an alternative therapy for patients intolerant to standard treatments, they have not yet replaced chemotherapy as the optimal approach for low-risk GTN. Future studies might explore ICIs as first-line treatments for selected low-risk GTN patients. This trial marks a significant step in expanding immunotherapy options for GTN beyond high-risk cases.

### 4.2 Challenges of ICIs in high-risk and chemoresistant GTN

Patients with multidrug-resistant GTN historically face a poor prognosis, but pembrolizumab has shown potential for achieving complete remission in this challenging group. The initial report in 2017 highlighted four patients with high-risk GTN treated after multiple chemotherapy regimens, resulting in three complete responses and one death (30). These promising outcomes led to the development of UK guidelines recommending pembrolizumab for high-risk GTN patients who have failed at least two lines of multi-agent chemotherapy, including EMA/CO, and as an alternative for PSTT/ETT cases post-platinum-based chemotherapy (41). During the COVID-19 pandemic, the guidelines were adjusted to permit pembrolizumab use after first-line EMA/CO. Treatment generally continues until hCG normalization or radiological disease resolution, followed by consolidation therapy. Some case reports further support pembrolizumab’s efficacy, showing hCG normalization with multidrug-resistant GTN (42–45).

Despite the potential benefits of ICIs, challenges remain, particularly in high-risk and chemoresistant GTN cases. Cohort B of the TROPHIMMUN Phase II Trial evaluated Avelumab in such patients, revealing mixed results. In this cohort, seven patients with polychemotherapy-resistant GTN received Avelumab at 10 mg/kg bi-weekly until hCG normalization, followed by three consolidation cycles. Only one patient (14.3%) achieved hCG normalization, highlighting limited efficacy, with a median resistance-free survival of 1.4 months and 57.1% of patients showing resistance to Avelumab (38). Although there are no head-to-head comparisons of anti-PD-1 and anti-PD-L1 agents, a meta-analysis showed that anti-PD-1 is more effective than anti-PD-L1 (46). This underscores the difficulty in overcoming the aggressive nature and chemoresistance of advanced GTN, pointing to the need for continued research and combination therapy approaches to enhance treatment outcomes.

The CAP01 study provided further insights into the application of PD-1 inhibitors and antiangiogenic agents in high-risk GTN. In the trial, 20 patients with high-risk, chemorefractory, or relapsed GTN were treated with camrelizumab (200 mg bi-weekly) plus apatinib (250 mg daily). The primary endpoint, objective response rate (ORR), was met by 55% of participants (95% CI 32–77), with 10 patients (50%) achieving complete response (CR) and no relapses after treatment discontinuation. Among the 10 patients who did not achieve CR, 7 responded effectively to salvage chemotherapy. The median time to CR was 3 cycles. The safety profile was considered manageable, with grade 3 TRAEs such as hypertension (25%), rash (20%), and neutropenia (10%). One patient discontinued treatment due to a serious adverse event (47). These results suggest that the combination of camrelizumab and apatinib demonstrates promising antitumor activity and could be explored as a salvage therapy for this challenging patient group. These findings indicate that while ICIs can contribute to disease control, they may not be sufficient as standalone treatments for high-risk or chemoresistant GTN.

A retrospective multicenter study assessed the safety and efficacy of combining anti-PD-1 therapy with chemotherapy versus anti-PD-1 monotherapy in patients with high-risk, chemorefractory, or relapsed GTN (48). Conducted across three hospitals in China, the study enrolled 66 patients, with 35 receiving anti-PD-1 therapy alone and 31 undergoing combination treatment. The ORR in the combined treatment group was significantly higher at 96.8% compared to 62.9% in the monotherapy group (p < 0.001). The CR rate was also greater in the combination group (87.1%) than in the monotherapy cohort (54.3%) (p = 0.007). The median time to achieve CR was 2.8 months for the combined group and 2.2 months for monotherapy. Progression-free survival (PFS) significantly favored the combined therapy group [HR 0.06 (95% CI 0.02–0.16), p < 0.001], while overall survival (OS) did not show a significant difference between groups [HR 0.50 (95% CI 0.07–3.24), p = 0.499]. TRAEs were comparable between groups, occurring in 77.1% of monotherapy and 80.6% of combination therapy patients, with the most common grade 3–4 events including leukopenia and elevated alanine aminotransferase levels. These results suggest that combining anti-PD-1 therapy with chemotherapy provides a significant benefit in ORR and PFS, offering an effective treatment option for high-risk GTN cases.

The ongoing exploration of combination therapies that integrate ICIs with conventional chemotherapy or targeted agents may hold promise for improving ORR. Current studies and anecdotal evidence suggest that such approaches might enhance the immune response and overcome resistance mechanisms inherent in high-risk GTN. However, these strategies require further validation through larger, prospective clinical trials to establish efficacy, safety, and optimal treatment protocols.

### 4.3 Role of ICIs in rare types of GTN

The role of ICIs in rare types of GTN, such as PSTT and ETT, has gained attention due to the unique challenges these tumors present. PSTT and ETT are known for their chemoresistance and slower growth, producing lower hCG levels relative to tumor volume. This profile limits the effectiveness of conventional chemotherapy and complicates treatment strategies. While the FIGO scoring system is not applicable to PSTT and ETT, treatment decisions are guided by specific prognostic factors, including an interval of over 48 months from the antecedent pregnancy and advanced stage IV disease, which are associated with poorer outcomes (17, 49).

Pembrolizumab has shown promise in treating chemo-resistant PSTT and ETT, as evidenced by multiple case reports (30, 50–52). For instance, the above case reports in 2017, three out of four patients with high-risk GTN, including 2 cases of PSTT and ETT, achieved complete remission following pembrolizumab treatment after failure of multi-agent chemotherapy (30). The TROPHIMMUN Phase II trial’s Cohort B, due to their pathological subtypes (4 patients with choriocarcinomas, 1 with ETT, 1 with PSTT, 1with an unknown subtype) and their FIGO scores at inclusion (≥ 10 for 4 of them), showed that while Avelumab led to hCG normalization in one out of seven patients (14.3%), its limited success underscores the complexity of treating chemoresistant GTN with monotherapy (38). Nonetheless, challenges remain, as the treatment of rare types like PSTT and ETT often requires aggressive multimodal approaches, including surgery and tailored combination regimens. These findings stress the need for further research to optimize the use of ICIs and combination therapies in rare GTN subtypes to improve patient outcomes.

### 4.4 Biomarkers and predictors of response to immunotherapy

The clinical studies highlighted above demonstrate variability in the response of GTN patients to ICIs. The success of ICIs in treating GTN largely hinges on the identification of reliable biomarkers to predict patient outcomes. This is particularly crucial given the heterogeneous nature of GTN. Recent advancements in genomic, transcriptomic, and tumor microenvironment analyses have shed light on potential biomarkers that could enhance the predictive accuracy for ICIs efficacy in GTN.

PD-L1 expression has been widely studied as a potential biomarker for immunotherapy, although its predictive value remains limited. Immunohistochemical studies reveal that PD-L1 is highly expressed in trophoblastic cells of GTN, including syncytiotrophoblasts and cytotrophoblasts, akin to its role in placental immune tolerance (29). Studies, such as the KEYNOTE-001 and KEYNOTE-052, have demonstrated a correlation between higher PD-L1 expression and improved clinical outcomes (53, 54). However, the heterogeneity of PD-L1 expression across tumor subtypes and the lack of standardized testing protocols complicate its application. Emerging evidence suggests that PD-L1 expression alone may not suffice as a standalone predictor, underscoring the need forcomplementary biomarkers.

Tumor mutational burden (TMB), defined as the number of somatic mutations per megabase, has been associated with responses to ICIs in various cancers. Studies, such as the KEYNOTE-158 trial, have shown that patients with high TMB (≥10 mutations/megabase) exhibit improved outcomes with pembrolizumab, leading to Food and Drug Administration (FDA) approval for its use in TMB-high solid tumors (55). However, the utility of TMB is limited by technical and biological factors. Technical limitations include variability in sequencing platforms and methodologies, while biologically, the predictive accuracy of TMB is affected by spatial and temporal tumor heterogeneity, subclonal variations, and the differing immunogenicity of individual mutations (56). In the case of GTN, however, TMB is generally low. A study analyzing 30 cases of GTN, including choriocarcinoma, ETT, PSTT, found that 92.8% of GTN samples exhibited low TMB. Despite this, some GTN subtypes, such as choriocarcinoma, showed changes in the homologous recombination repair (HRR) genes (57). These findings highlight that although TMB may not be a robust predictive biomarker for immunotherapy in GTN, other genetic and molecular features may provide useful insights for identifying patients who could benefit from ICIs.

Microsatellite Instability (MSI) and Mismatch Repair Deficiency (dMMR) are well-established biomarkers for predicting response to ICIs in multiple cancers. MSI, characterized by insertions or deletions in microsatellite sequences, emerges due to deficiencies in the MMR pathway (58, 59). These deficiencies result in genomic hypermutability, increased neoantigen production, and an immune-rich tumor microenvironment (TME), making MSI-high (MSI-H) tumors responsive to ICIs (60–62). The KEYNOTE-016 trial demonstrated an ORR of 40% for pembrolizumab in dMMR/MSI tumors, leading to FDA approval (63). Subsequent studies, like KEYNOTE-177 and CheckMate-142, evaluated pembrolizumab and nivolumab in metastatic colorectal cancer, reporting median PFS improvements and durable responses (64, 65). Furthermore, dual-agent approaches combining PD-1 and cytotoxic T lymphocyte-associated antigen 4 (CTLA-4) inhibitors show potential, as seen in CheckMate-142, which reported a 55% ORR in dMMR/MSI metastatic colorectal cancer (65). However, the prevalence of MSI-H or dMMR in GTN subtypes is not well-characterized, and further research is needed to explore their role in guiding immunotherapy.

TME plays a pivotal role in determining the response to ICIs. The presence of tumor-infiltrating lymphocytes (TILs), particularly CD8+ cytotoxic T cells, correlates with better responses to ICIs (66). In the context of GTN, a study demonstrated that TILs are associated with improved responses to pembrolizumab, further emphasizing the importance of TILs in immune response modulation (30). Conversely, immunosuppressive cells like Tregs and myeloid-derived suppressor cells (MDSCs) may limit the efficacy of ICIs by creating an anti-inflammatory TME (67, 68). Additionally, T-cell receptor (TCR) diversity and clonality measured through sequencing offer insights into immune activation and responsiveness to ICIs (69). High TCR clonality has been associated with better outcomes in head and neck squamous cell carcinoma and may hold promise in GTN (69). Furthermore, HLA-G expression has been identified as a potential biomarker for predicting response to ICIs in GTN, as it plays a role in immune evasion by modulating NK and T cell activity (30). This highlights the complexity of immune interactions within the TME and the potential for targeting multiple immune pathways to enhance therapeutic responses in GTN.

Despite promising biomarkers, resistance to ICIs remains a challenge in GTN. Mechanisms such as PD-L1 loss, TME immunosuppression, and alternative immune checkpoint activation (e.g., B7-H3 and VISTA) contribute to primary and acquired resistance (70). Understanding these resistance pathways is crucial for developing combination therapies that enhance ICIs efficacy. Integrating multiple biomarkers, such as PD-L1, TMB, MSI, and TME, could improve patient stratification and treatment personalization in GTN. Prospective studies combining ICIs with targeted agents like VEGF inhibitors may offer synergistic effects, overcoming resistance mechanisms. Further, advancements in single-cell sequencing and spatial transcriptomics will provide deeper insights into the interplay between tumor genetics and the immune microenvironment, paving the way for precision immunotherapy in GTN.

## 5 IrAE of ICIs

IrAEs are a critical consideration when using ICIs for cancer treatment, including in the context of GTN. While ICIs have revolutionized cancer treatment by enhancing immune responses against tumors, they also lead to unintended activation of the immune system, which can result in damage to normal tissues and organs (71–79). These immune-mediated side effects present a significant challenge in the clinical use of ICIs, especially in diseases like GTN, where the patient population is often young and reproductive health is a major concern.

### 5.1 Variation in irAE across ICI types and cancers

The types of irAEs vary depending on the specific ICIs used. A meta-analysis of 48 trials found that PD-1 blockade is associated with a higher frequency of pneumonitis, myalgia, hypothyroidism, arthralgia, and vitiligo, whereas CTLA-4 blockade tends to result in more colitis, hypophysitis, and skin-related irAEs (80). Additionally, even when the same ICI is used, the incidence of irAEs differs slightly across different diseases. When comparing melanoma, non-small cell lung cancer (NSCLC), and renal cell carcinoma, melanoma patients tend to experience more gastrointestinal and skin-related irAEs, whereas pneumonitis is more common in those with NSCLC and renal cell carcinoma (80). Based on clinical trial data of ICIs in GTN, the occurrence of irAEs has also shown to affect various systems (37, 47).

### 5.2 Spectrum of irAE across organ systems

The spectrum of irAEs associated with ICIs is broad and can involve virtually any organ system (80). Some of the most common irAEs are dermatologic, including pruritus, rashes, and vitiligo, as well as more severe manifestations like blistering or erythema multiforme (81). Gastrointestinal toxicity, particularly diarrhea, colitis, and abdominal pain, can occur and, in severe cases, may result in life-threatening conditions like colonic perforation (82, 83). Endocrinopathies are also common, with thyroiditis (hypo- or hyperthyroidism) being the most frequently reported, though adrenalitis, hypophysitis, and diabetes have also been observed (84–86). Hepatic toxicity, characterized by elevated liver enzymes (AST, ALT), may progress to immune-mediated hepatitis and, in rare cases, liver failure (76). Pulmonary complications, such as pneumonitis, and cardiovascular effects, including myocarditis, are less common but can be fatal if not promptly recognized and treated (73, 79).

As more patients achieve long-term survival with ICIs, the significance of chronic irAEs is increasingly recognized. Chronic irAEs, defined as lasting beyond 3 months after treatment discontinuation, affect 43% of patients in certain studies, with mild cases being most common (87, 88). These chronic events include endocrinopathies, arthritis, xerostomia, neurotoxicities, and ocular issues, and are often irreversible (88). Though these irAEs can impact quality of life, particularly in long-term survivors, they are underreported in clinical trials.

Fatal irAEs, though rare, occur in up to 2% of patients, with severe cases like myocarditis and pneumonitis being most lethal (89). Additionally, long-term cardiovascular risks, such as increased aortic plaque volume and higher incidence of myocardial infarction, are emerging as significant concerns (90). Studies suggest T-cell mediated inflammation may drive ICI-related atherosclerosis, highlighting the need for proactive cardiovascular surveillance and management strategies for ICI-treated patients (91, 92).

### 5.3 Management of irAE

While ICIs offer promising benefits in treating GTN, the potential for irAEs presents a significant challenge to treatment. Grade 3 or higher irAEs generally require immunosuppressive treatment. The first-line treatment is usually discontinuation of the ICI therapy, followed by the administration of corticosteroids (93–95). High-dose corticosteroids are generally effective and are typically initiated at doses ranging from 0.5 to 2 mg/kg of prednisolone (93–95). In cases of severe irAEs, such as myocarditis or pneumonitis, higher doses of corticosteroids, including methylprednisolone (1,000 mg/day), may be required (93–95). Steroid treatment usually leads to symptom improvement within days, and after resolution, steroids are gradually tapered to minimize long-term side effects (96). However, the prolonged use of corticosteroids carries its own risks, such as increased susceptibility to infections and potential effects on tumor immunity. For patients who do not respond to corticosteroids or experience relapse of symptoms, second-line immunosuppressive agents may be required. These include biologics such as infliximab (anti-TNF-α) for gastrointestinal irAEs like colitis (96), and other immunosuppressive agents like methotrexate for managing severe autoimmune reactions (97). The choice of second-line therapy is often guided by the severity of the irAE and requires careful monitoring.

When these treatments effectively manage irAEs, they also pose a risk of impairing the anti-tumor immune response. Some studies indicate that low-grade irAEs may provide a more pronounced survival benefit, while high-grade irAEs, often requiring immunosuppressive treatment, might compromise the efficacy of ICIs (98, 99). The effects of combined immunosuppression and ICIs are inconsistent due to study design variations. For example, tumor necrosis factor (TNF) blockade may enhance ICI efficacy by supporting tumor-infiltrating lymphocyte survival (100, 101), while IL-6 receptor blockade could induce remission without impairing anti-tumor immunity (100, 102, 103). However, corticosteroids and second-line immunosuppressants might reduce ICI effectiveness (104). While ongoing clinical studies aim to provide clarity, current research is often limited by insufficient statistical power to accurately assess ICI responses. To optimize outcomes, strategies involving meticulous monitoring, personalized treatment plans, and regular organ function evaluations are essential for maintaining patient safety and maximizing therapeutic efficacy.

## 6 ICIs on fertility

As GTN primarily affects patients of childbearing age, and hysterectomy is generally not required for types other than PSTT and ETT, preserving fertility should be considered before planning treatment. Therefore, understanding the potential impact of immunotherapy on patients’ fertility and fetal health is particularly important.

### 6.1 Impact of ICIs on reproductive health

ICIs alter immune responses by reactivating T cells to target cancer cells. However, this broad immune activation can have unintended consequences on gonadal function. Preclinical studies have shown that ICIs increase immune cell infiltration and cytokine levels, such as TNF-α and IL-1β, within ovarian tissues, leading to follicular atresia in mouse models (105). Furthermore, ICIs disrupt the hypothalamic-pituitary-gonadal axis through endocrine irAEs such as hypophysitis, which can result in reduced gonadotropin levels and secondary hypogonadism (106). The PD-1/PD-L1 and CTLA-4 pathways, essential for maternal-fetal tolerance, are also implicated in reproductive health. By blocking these checkpoints, ICIs interfere with immune-regulatory mechanisms essential for implantation and pregnancy maintenance, thereby increasing risks of implantation failure and pregnancy complications (107). Animal studies reinforce these findings, showing that treatment with anti-PD-L1 antibodies or nivolumab in pregnant mice and cynomolgus monkeys led to significantly higher abortion rates and premature neonatal deaths, primarily through the depletion of regulatory T cells (108).

### 6.2 Outcomes of ICIs use during pregnancy

Clinical data further illustrate the complexities of using ICIs during pregnancy. Seven case reports document outcomes of women who either conceived while on ICIs or initiated treatment during pregnancy, including two twin pregnancies (52, 109–114). These cases predominantly involved patients with metastatic melanoma, with one exception being a PSTT case treated with pembrolizumab. Treatments included nivolumab (alone or with ipilimumab), pembrolizumab, and ipilimumab with intralesional IL-2. Pregnancy complications were notable, with four cases of intrauterine growth restriction or placental insufficiency leading to preterm caesarean sections (109–112). Among nine neonates, one exhibited transient congenital hypothyroidism, and another was born without a left hand, because of strangulation by the amniotic cord unrelated to the immunotherapy (110). At follow-up (6 weeks to 2.75 years), all children were healthy with normal developmental milestones. Maternal outcomes varied; while three mothers experienced disease progression (111, 113, 114), the remaining four patients achieved remission or stable disease during pregnancy. These cases underscore the challenges of balancing cancer treatment with pregnancy management, showing generally favorable neonatal health but variable maternal outcomes.

### 6.3 Post-treatment fertility and pregnancy outcomes after ICIs

Building on these findings, a recent retrospective cohort study further examined the post-treatment impact of ICIs on fertility and pregnancy outcomes in 53 reproductive-age patients treated for various cancers (115). Among these, 15% (8 patients) successfully conceived post-treatment after a median interval of 10.5 months (range 2-30 months), highlighting the possibility of fertility preservation despite prior immunotherapy. Of these pregnancies, five were carried to term, with minimal complications; however, one patient experienced a first-trimester miscarriage followed by a successful pregnancy. Maternal complications included cases of gestational diabetes and preeclampsia, attributed to pre-existing risk factors rather than ICIs. Importantly, all neonates were born healthy, with normal growth and developmental milestones reported during follow-ups. Beyond pregnancy outcomes, the study also explored ICI-induced endocrine disruptions, noting a significant decline in anti-Müllerian hormone (AMH) levels in one patient, suggesting potential long-term impacts on ovarian reserve. Preclinical evidence aligns with these findings, demonstrating ICI-associated gonadotoxicity, including immune cell infiltration, cytokine-mediated ovarian damage, and diminished follicular reserves in animal models (105, 116, 117).

### 6.4 Fertility preservation strategies in patients receiving ICIs

Given the potential risks to fertility associated with ICI therapy, fertility preservation has become an essential consideration for reproductive-age patients. Cryopreservation remains the cornerstone of fertility preservation strategies (118–120). Oocyte and embryo freezing, typically performed after controlled ovarian stimulation, are the most established methods. For patients who require immediate cancer treatment and cannot delay therapy, ovarian tissue cryopreservation offers a viable alternative (118–120). Pharmacological approaches, such as gonadotropin-releasing hormone agonists (GnRHa), have been employed to suppress ovarian function and potentially reduce gonadotoxicity during chemotherapy. However, their efficacy in the context of ICI therapy is not yet well established, requiring further research to determine their protective role. Emerging techniques, such as *in vitro* follicle maturation (IVFM) and regenerative therapies like mesenchymal stem cell-derived exosomes, show promise for restoring ovarian function and repairing gonadal damage caused by immunotherapy (120). These advancements, alongside comprehensive fertility counseling and a multidisciplinary approach involving oncologists, endocrinologists, and reproductive specialists, are essential for optimizing fertility preservation strategies and aligning treatment plans with patients’ reproductive goals.

## 7 Summary and future prospects

Immunotherapy has emerged as a transformative approach in the treatment of GTN, particularly for patients with chemoresistant or high-risk disease. Recent trials, such as the TROPHIMMUN study, have demonstrated the potential of ICIs like avelumab in low-risk GTN patients resistant to MTX, with promising outcomes including a 53% CR rate. Similarly, the CAP01 study highlighted the efficacy of combining camrelizumab with apatinib, achieving a CR rate of 50% in patients with chemorefractory GTN. These results underscore the potential of ICIs, both as monotherapies and it combining with other therapeutic agents, to overcome the challenges of chemoresistance in GTN.

Looking forward, several ongoing clinical trials are further investigating the role of ICIs in GTN (**Table 2**), with a particular focus on combination therapies. The RESOLVE study (NCT05635344) warrants further discussion as an example of attempts to use immunotherapy earlier in the disease course and limit exposure to chemotherapy. The TROPHAMET Phase I/II trial (NCT04396223) is examining the combination of avelumab with MTX as a first-line treatment for low-risk GTN. This study aims to determine the effectiveness of this combination in treating chemotherapy-resistant cases of GTN. The study, reported at ESMO 2024, has shown promising early results, with a high rate of successful hCG normalization, indicating that the combination of avelumab and MTX might improve treatment outcomes compared to single-agent chemotherapy. With a median follow-up of 24.8 months, the trial reports a 96.2% success rate in achieving hCG normalization and suggests that this combination therapy may provide long-term remission for low-risk GTN patients. Similarly, the CR-GTP trial (NCT04303884) is assessing the clinical efficacy of pembrolizumab in patients with GTN resistant to multi-agent chemotherapy.

**Table 2:** Ongoing studies focusing on immunotherapy for gestational trophoblastic neoplasia.

Identifier (name)/Years	Type of study	Agents	Estimated enrollment	Participants	Primary endpoint	Estimated completion date
NCT04396223(TROPHAMET)/2020	Phase I/II	Avelumab and methotrexate	26	low-risk GTN not treated before	1. Incidence of dose limiting toxicities of methotrexate and avelumab combination 2. Rate of patients with successful hCG normalization	October 2028
NCT04303884(CR-GTP)/2020	Phase II	Pembrolizumab	15	Histologically confirmed diagnosis of GTN refractory or chemo-resistant to multi-agent chemotherapy	1. Radiological response according to iRECIST criteria 2. Serologic response assessed by serum b-hCG	May 2023
NCT04812002/2021	Phase II	PD-1 inhibitor and bevacizumab	20	Relapsed or refractory high-risk GTN after second line or above combined chemotherapy	Progression-free survival	April 2026
NCT05139095/2022	Phase II	Camrelizumab and apatinib and chemotherapy	73	Ultrahigh-risk or highrisk chemo-refractory or relapsed GTN	1.Complete remission rate 2.Objective response rate	June 2024
NCT06028672/2023	NA	Experimental: Toripalimab and actinomycin-D Control: actinomycin-D	40	GTN with FIGO score 5-6 not treated before	Complete remission rate	August 2025
NCT06020755/2023	Phase II	Toripalimab and actinomycin-D	17	GTN with FIGO score 7 not treated before	Complete remission rate	August 2025
NCT05635344(RESOLVE)/2024	Phase II	Experimental: Pembrolizumab and second evacuation Control: second evacuation only	20	Low risk postmolar GTN after primary surgical evacuation with no intervening treatment	The feasibility of conducting a definitive study of neoadjuvant pembrolizumab prior to second evacuation of low risk postmolar GTN	August 2026

GTN, gestational trophoblastic neoplasia;, hCG, human chorionic gonadotropin; iRECIST, immune response evaluation criteria in solid tumors; PD-1, programmed cell death protein 1; NA, not available; FIGO, the international federation of gynecology and obstetrics.

Two other studies are evaluating the combination of Toripalimab and ActD in GTN patients with FIGO scores of 5-6(NCT06028672) and 7(NCT06020755), respectively, offering new possibilities for integrating ICIs into standard regimens. Another combination therapy study (NCT04812002) focuses on patients with relapsed or refractory high-risk GTN who have previously undergone second-line or higher chemotherapy. The study aims to evaluate the PFS in patients receiving PD-1 inhibitors in combination with bevacizumab. Additionally, the combination of camrelizumab and apatinib is currently being tested in a CAP01for patients with ultra-high-risk or chemorefractory GTN, following encouraging results from earlier studies. This trial (NCT05139095), which started in January 2022, is expected to provide valuable insights into the potential of combining ICIs with anti-angiogenic therapies in managing aggressive forms of GTN.

Biomarker-driven approaches are also gaining traction. Advances in molecular profiling have identified potential predictive biomarkers such as PD-L1, TMB, MSI expression, and TILs. Furthermore, next-generation sequencing of GTN subtypes like choriocarcinoma, PSTT and ETT is uncovering specific genetic mutations that may guide personalized immunotherapy​. Challenges remain, including the need for more robust clinical evidence and the management of irAEs. The high cost of ICIs and accessibility issues also limit their widespread adoption. Future clinical trials must address these barriers while assessing long-term outcomes such as PFS, OS, and fertility preservation.

In conclusion, while immunotherapy, particularly ICIs, shows great promise in treating GTN, there is still much to learn. Ongoing clinical trials and international collaborations will be critical in advancing our understanding of how to optimize immunotherapy for GTN, overcoming current limitations, and ensuring that these therapies are accessible and effective for a broader range of patients. As more data emerges, it is likely that immunotherapy will become an integral part of the treatment paradigm for GTN, alongside conventional chemotherapy and surgery, improving survival and quality of life for patients with this rare and challenging disease.
